# Barriers to data quality resulting from the process of coding health information to administrative data: a qualitative study

**DOI:** 10.1186/s12913-017-2697-y

**Published:** 2017-11-22

**Authors:** Kelsey Lucyk, Karen Tang, Hude Quan

**Affiliations:** 1 Department of Community Health Sciences, Cumming School of Medicine, University of Calgary, 3rd Floor TRW, 3280 Hospital Drive NW, Calgary, Alberta, T2N 4Z6 Canada; 2 Department of Medicine, Cumming School of Medicine, University of Calgary, Health Sciences Centre, Foothills Campus, 3330 Hospital Drive NW, Calgary, Alberta, T2N 4N1 Canada

**Keywords:** Abstracting, Administrative data, Health information, Informatics, Qualitative research

## Abstract

**Background:**

Administrative health data are increasingly used for research and surveillance to inform decision-making because of its large sample sizes, geographic coverage, comprehensivity, and possibility for longitudinal follow-up. Within Canadian provinces, individuals are assigned unique personal health numbers that allow for linkage of administrative health records in that jurisdiction. It is therefore necessary to ensure that these data are of high quality, and that chart information is accurately coded to meet this end. Our objective is to explore the potential barriers that exist for high quality data coding through qualitative inquiry into the roles and responsibilities of medical chart coders.

**Methods:**

We conducted semi-structured interviews with 28 medical chart coders from Alberta, Canada. We used thematic analysis and open-coded each transcript to understand the process of administrative health data generation and identify barriers to its quality.

**Results:**

The process of generating administrative health data is highly complex and involves a diverse workforce. As such, there are multiple points in this process that introduce challenges for high quality data. For coders, the main barriers to data quality occurred around chart documentation, variability in the interpretation of chart information, and high quota expectations.

**Conclusions:**

This study illustrates the complex nature of barriers to high quality coding, in the context of administrative data generation. The findings from this study may be of use to data users, researchers, and decision-makers who wish to better understand the limitations of their data or pursue interventions to improve data quality.

**Electronic supplementary material:**

The online version of this article (10.1186/s12913-017-2697-y) contains supplementary material, which is available to authorized users.

## Background

Administrative health data refers to the information that is collected within the health-care system for reasons other than clinical care [1]. Some examples include physicians’ billing claims, pharmacy claims, and the hospital discharge abstract database (DAD). While collected primarily for administrative purposes, administrative health data are increasingly used for health research because of their possibility for longitudinal follow-up, large sample sizes, and wide geographic coverage of populations [2]. Given its accessibility, cost effectiveness, and comprehensive capture of episodes of health care contact, administrative data are also used for population health surveillance, evaluation of the quality of healthcare delivery, and to inform policy-related issues [3–6].

In light of its increased use, it is therefore necessary to ensure that administrative health data are of high quality [2]. Some recent studies have examined the role of abstracting (i.e., translating or coding information from the patient encounter into data) on data accuracy [7–12], while others have focused on the factors that influence medical abstracting and data quality, particularly concerning the completeness and consistency of documentation by healthcare providers [13–18]. Among studies that interrogate the quality of administrative health data, few seek to understand the role of coders (i.e., the health information professionals who abstract data) within the process of administrative data generation. Narus et al. (2011), for example, illustrate the process of data flow for the Pediatric Health Information System, but begin with the data sources that constitute this system and do not delve into the specifics regarding how they are generated [19]. The Canadian Institute for Health Information (CIHI) provides well-illustrated descriptions of data flow and generation [20]. Yet, CIHI focuses on errors that exist in the data after coders have submitted their abstracted data (e.g., missing data) [21, 22]; thus there remains the need to identify and understand the issues that exist for coders which lead to these data errors. Another study, by O’Malley et al. (2005), examined sources of errors throughout the in-patient coding process, from patient admission to discharge along the paper trail [23]. While this work provides a valuable overview of the inpatient coding process and potential sources of errors, it is specific to the American setting and the findings are not informed by the perspectives coders [23]. One study conducted in the Canadian context, by Hennessy et al. (2010), re-abstracted charts from Calgary-based coders and linked the findings to coders’ employment status, hospital level, and volume of coding, among other factors [24]. The authors found no consistent patterns between coder characteristics and face validity indicators (e.g., number of diagnoses coded) [24]. As such, there remains the need to identify influences on the quality of medical chart coding from the perspectives of those responsible for its abstraction.

**Fig. 1:**
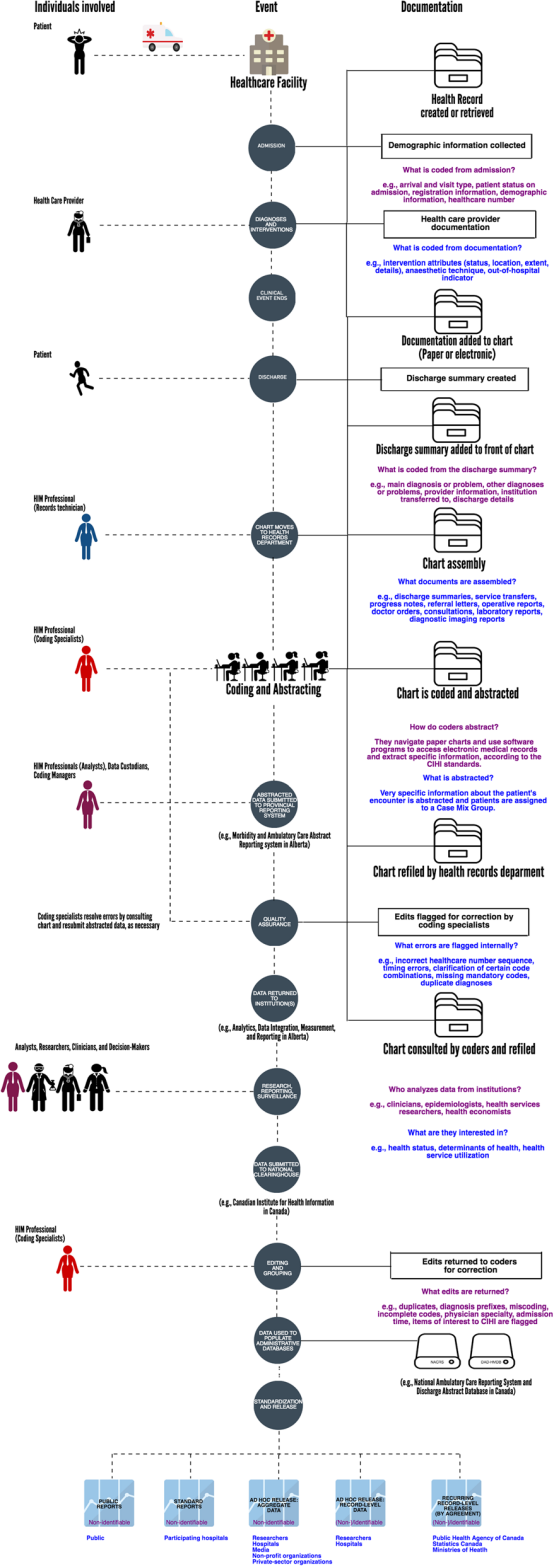
Process of administrative data generation

Our objective is to explore the potential barriers that exist for the high quality coding of administrative health data, through qualitative inquiry into the roles and responsibilities of Canadian medical chart coders. To situate our findings in the Canadian context of administrative health data, we explain and illustrate the process of administrative data generation from hospital and emergency department medical charts, drawing on examples from Alberta, in supplementary files (Fig. 1 and Additional file 1).

## Methods

We (KL and KT) interviewed 28 accredited health information management professionals (“coders”) who worked across Alberta, in both rural and urban settings. At the time of data collection, KL was a PhD candidate and research associate and KT was a physician (MD) and clinical research fellow; both investigators are female. We recruited participants first through purposive sampling by contacting coders using the information they listed on their professional networks (i.e., LinkedIn and the [former] Health Information Management Association of Alberta). No previous relationship was established with participants prior to recruitment. During our recruitment email or telephone call, we informed potential participants of the aims of our study and how we hoped their insights may improve data quality. After a first round of interviews, we used snowball sampling [25] to recruit the remainder of our sample. We received feedback on our interview guide prior to recruitment from 3 of our departmental colleagues who work with coded health information; one of these colleagues participated in an unrecorded pilot interview.

Interviews were conducted between September 2015 and March 2016. Each semi-structured interview was conducted in-person at our research institution or by telephone and was approximately 1 h in length. Both investigators recorded field notes during the interview process. We audio-recorded and transcribed interviews, which focused on areas where the literature had suggested barriers to medical chart abstraction: training, documentation, CIHI standards, classification systems, workload, work environment, and collaboration. We prompted participants with follow up questions for clarity and further details throughout each interview. Our interview guide is provided in Additional file 2. No repeat interviews were conducted and transcripts were not returned to participants for comment. We used NVivo 11 to manage our data.

We employed thematic analysis to analyze transcripts in their anonymized form [26], with each KL and KT independently open-coding line-by-line, with no pre-defined framework. The work experience of coders ranged from 1.5 to 37 years, with an age range of 22 to 71 years. The majority of coders worked full-time, with some working part-time, casually, or temporarily. Our sample included 3 males and 25 females. To fully understand the breadth of coded health information within the context of its use, we also interviewed 3 data users who work daily with coded health information.

As codes were generated and themes emerged, we re-analyzed transcripts through constant comparison. The authors met throughout data collection and analysis to discuss emergent themes and data saturation. Data saturation occurred after approximately 20 interviews; however, as both data analysis and collection occurred simultaneously we completed our scheduled interviews, resulting in our sample of 28 coders, and used these to further refine our themes.

For this paper, we focused our analysis on areas that shed light on the process of data generation for medical chart coders and identifying potential barriers to high data quality might be influenced at the various levels in this process.

## Results

Throughout the process of administrative data generation, there are a number of instances where potential exists to adversely influence data quality. From the perspective of coders, four areas emerged as presenting particular challenges: the documentation of clinical events from admission to discharge; chart organization and assembly; variability in interpretation; and, quotas and expectations.

### Documentation of clinical events from admission to discharge

The documentation that healthcare providers complete to describe a patient’s encounter with the healthcare facility is perhaps the resource most influential to data quality, as it constitutes the information that is interpreted by coders, abstracted, and sent to institutional repositories. As such, issues that occur upstream in the process of data generation have the potential to adversely influence data quality downstream for coders, researchers and analysts, and decision-makers.

It should be noted that while all of these sources are used to *inform* coding specialists’ abstracting, they are not assessed equally. Coders have standards and guidelines administered to them by CIHI about which documents are permitted to be used and how. For instance, when abstracting for the DAD, coders are required to assign diagnosis types to all significant/comorbid diagnoses and conditions: most responsible diagnosis (type M), comorbidity diagnoses (types 1 and 2), proxy most responsible diagnosis (type 6), and service transfer diagnoses (types W, X, and Y) [27]. For example, coders *must* use physician documentation (e.g., discharge/case summary, report of history and physical exam, progress notes, consultation reports, intervention reports, diagnostic imaging reports) to assign diagnosis types to conditions; however, documentation by other health care providers (e.g., midwife, nurse practitioners) can also be used with limitations [27]. Comorbidities, for instance, are assigned a diagnosis type 1 (i.e., pre-admit comorbidity) or 2 (i.e., post-admit comorbidity) if the physician has documented the coexisting condition and it also meets the criteria for significance (i.e., requires treatment beyond maintenance of the condition, increases length of stay by at least 24 h, or significantly affects the treatment) [27]. If a physician has not documented the coexisting condition but other healthcare providers have, it is assigned a diagnosis type 3 (i.e., secondary diagnosis) and not considered significant. Additional coding rules apply for abstracting hospital-based and community-based ambulatory care using the National Ambulatory Care Reporting System Metadata (NACRS). Differences between the DAD and NACRS are provided in Additional file 3: Table S1.

However, the primary purpose and function of documentation as a record of patient treatment and care presents a fundamental issue to its secondary use for medical abstraction. First, physicians document their procedures and diagnoses in ways that are standard within the healthcare profession. For instance, they may document quickly and efficiently by providing brief but informative descriptions, which serve the purpose of patient care but provide challenges for coders who are required to provide further details when abstracting. For example, physicians may not document specific details of an intervention (e.g., length of a laceration or use of anesthetic) or diagnosis (e.g., pancreatitis without specifying whether it was acute, idiopathic, alcohol-induced, or sclerotic) if it is not deemed necessary to inform a patient’s treatment. In other cases, providers may not list a definitive diagnosis or may provide details of a diagnosis without specifying the condition. This presents a challenge to coders, whose standards dictate that diagnoses must be coded only from sources where physicians have explicitly documented it. For example, a physician may list a glomerular filtration rate to indicate the stage or severity of chronic kidney disease; however, coders are not permitted to interpret that range (even if they are able to identify it correctly) and therefore must code it as ‘chronic kidney disease, unspecified.’ In such cases, coders attempt to glean further detail from other sources of documentation (see Table 1) to collect the detailed information they need.

**Table 1:** List of documentation sources coders reported using

Types of Documentation	
• Discharge summaries or ambulatory care records • Triage sheets • Transfer summaries • Service transfers • Progress notes (physician or nurses) • Nursing assessments • Operative reports or peri-operative reports • Referral letters • Consultations • Doctor orders	• Pathology reports • Laboratory test results • Microbiology reports • Diagnostic imaging reports • X-Ray reports • Radiology reports • Haemodialysis records • Transfusion records • Special procedures • Prescription orders • Patient histories • Patient physicals • Admission histories

A second issue concerns the terminology that physicians use, which again serves the purpose of patient care but presents a challenge to abstraction. For efficiency, physicians may use shortened terms or acronyms to refer to diagnoses or interventions, or may use colloquialisms in place of medical terminology for the same purpose, but coders are required to use ICD-10 classifications when abstracting chart information. If a physician uses diagnoses not found in ICD-10 or use non-standardized acronyms (e.g., “BIBA” to indicate “brought in by ambulance”) that coders may not familiar with, there is the potential for this information to be mistranslated. A further issue is introduced by the CIHI coding standards, which suggest that documentation occurs in a standardized way, although there are currently no standards in place in many institutions. In Alberta, physicians are required to complete a discharge summary “in a timely manner consistent with the policies of the institution,” with no further guidance outlined [28]. Finally, the CIHI standards suggest that coders should return charts to physicians for greater clarification where necessary; however, we found that this practice was strongly discouraged across urban sites. One participant shed light on this situation by acknowledging the frustration that physicians faced when asked to code more specifically and the frustration coders faced when trying to follow standards that physicians do not:

All [physicians] wanted to worry about was the patient and now they’re having to worry about how they document and what they write. We’re telling them: you call it *this*, but we need to hear *these words*. I mean, it’s a shame that if CIHI is making the rules that they are not teaching them, too [Interview 28].

A third, albeit less consequential, frustration that coders voiced as occurring during the documentation process was the legibility of written, paper documents. Coders understood that illegibility would inevitably occur some of the time, due to the fast pace of patient care and volume of cases physicians dealt with. However, they also noted that illegibility did influence coding. In most cases, coders could interpret handwriting by consulting their peers or gaining greater experience with certain physicians’ writing styles. Yet there were times when coders were required to apply their ‘best guess’ to deciphering documentation, which could result in the erroneous or imprecise capture of a diagnosis or procedure. It is worth noting that despite the ability of coders to overcome this barrier (e.g., consulting peers and other forms of documentation), the process of deciphering handwriting, searching for detail to support a procedure, or translating terminologies, results in time away from coding. Because of the pressure of coding quotas (see below), coders may simply adopt an unspecified code for a specific diagnosis or procedure where this information is not readily found. This contributes to frustrations experienced by researchers and analysts downstream, when they attempt to gain insight into questions concerning specific conditions or treatments. One coder spoke of how this may be particularly frustrating to clinician researchers, who do not understand how their documentation is interpreted and abstracted:

When [physicians] get the reports back from analysts and they realize the procedures they’re doing [are] not matching what is in the reports, then they realize there’s a big problem with the coding [Interview 27].

### Chart organization and assembly

Following a patient’s discharge, their chart is moved from the ward or clinic to a central facility (e.g., health records department) to be organized and assembled in preparation for coding and abstraction. The main challenge for data quality that is introduced at this stage relates to the completeness of charts when they are passed onto coders. Province-wide, Alberta follows a schedule whereby all abstracts from the present month must be submitted to Alberta Health Services on the 21st day of the next month (e.g., all April hospitalizations must be abstracted and submitted by May 21). This introduces pressure for health information management professionals (e.g., record technicians, coding managers, and coders) whose work is largely evaluated by their ability to meet this timeline. Healthcare providers, however, are not subject to the same pressures, which results in a delay between a patient’s discharge and the completion of a discharge summary by physicians. Sometimes physicians take up to 1 year to complete their discharge summaries; as such, record technicians compile charts for coders without the presence of this important document.

#### Incomplete chart documentation

Incomplete charts (i.e., missing the discharge summary or operative report) carries implications for data quality. As described by one coder, when faced with an incomplete chart, “We just have to virtually go through the chart and hope that we’re coding it correctly and there’s no doubt we’re missing things” [Interview 5]. Most coders recognized that incomplete charts degrade data quality, as one even explicitly stated, “With incomplete charts, you get incomplete data” [Interview 27]. However, coders face limited options to improve data quality; as their job requires them to meet their quota, they must use available documentation. Another coder went on to describe how without the discharge summary, coders are abstracting diagnoses and procedures based on incomplete — and possibly incorrect — information that was subject to change following their abstraction. As she stated:

…they’re telling me to code from the admit diagnosis. You know, from admit to the time they’re discharged is completely different… I can’t come in here anymore because I don’t feel comfortable because […] my coder number is on it [Interview 28].

Aside from the completion of charts, the turnaround time also adversely affected how charts were organized, which tended to delay the coding process. Some coders explained that the charts were no longer re-organized by records management clerks once received from the unit to save costs and improve efficiency. Consequently, this resulted in frustration and inefficiencies for coders. As one coder mentioned:

…sometimes things are not in order which is not a problem for most days […] But a chart that’s three volumes that’s not organized can… half our time can be consumed just trying to get everything in the right order so we can make sense of it [Interview 23].

Another coded recalled how, “The charts used to be rigorously assembled,” but that was found to be “pretty brutal for the assemblers because […the documents] had to be in a very specific order” [Interview 12]. This coder went on to say that once the changes were implemented by records management clerks, charts began to resemble a “dog’s breakfast” because the “people in assembly have been instructed to just strictly punch the holes [into chart documentation] and stuff them into folders” [Interview 12]. Another coder reinforced this point about disorganized charts, by sharing that:

They’re not in any order. When you’re looking at progress notes and you need to know was this a complication in a thirty-day time and the notes are not in any order, it slows you down quite a bit. Productivity can be affected by that [Interview 28].

In principle, coders are expected to return to an abstract once they receive the discharge summary to ensure that they have accurately captured the physician’s documented diagnoses and procedures. However, the sheer volume of charts that coders must abstract in a day (i.e., up to 30 for inpatient charts and up to 180 for ambulatory and emergency coding), along with the pressure to meet their quota, makes this an infeasible practice – especially considering that up to 80% of charts received are estimated as incomplete (i.e., without a discharge summary) [Interview 28]. This introduces the potential for miscoding data, whereby the conditions recorded by other healthcare providers (on admission and throughout the process) may differ from the doctor’s diagnosis and the disease’s progression at the time of discharge. While this represents a potential significant issue, the extent of this problem currently remains unknown.

### Variability in interpretation

One challenge that occurs at the level of coding with implications for data quality is the interpretation that coders must apply when they are uncertain how to capture certain documented (or undocumented) features. Regardless of the standards and procedures that coders systematically follow, some variability among coders is inevitable, due to unspecified documentation or unidentified diagnoses. As one site coordinator explained about coding physician documentation:

We try to interpret what they’re saying, trying not to do a lot of assuming, but then we also have our own standards. So trying to find a compromise between them… [Interview 20].

A common example of interpretability is illustrated through the coding of a urinary tract infection (UTI) and urosepsis. In one of their abstracting software (i.e., Folio), coders are directed to the same code for both of these conditions [Interviews 20, 24]. However, after communicating with physicians, a coordinator realized that urosepsis and UTI were not the same condition (i.e., urosepsis was sepsis with UTI) and should not be coded as such. Two sites responded to this issue differently. At one site, coders were instructed to code sepsis as the most responsible diagnosis to accurately reflect the high resource intensity weight and length of stay incurred by sepsis [Interview 20]. At another site, however, the coordinator instructed coders to code the most responsible diagnosis as a UTI, because “If you [meaning a doctor] meant sepsis, you better say sepsis” [Interview 25].

The above example also represents the wider issue of the regional coding variations that exist between sites across the province. One coder reflected on how the practice of capturing intravenous antibiotic differed between Edmonton and Calgary sites where she had worked. She noted that, “We wouldn’t code it up in Edmonton but they did in Calgary. Then I kind of went back and forth on whether it’s supposed to be coded or not because they [meaning the North Zone] also talked to the South Zone and they were doing it different […]” [Interview 21]. This practice introduces the potential for discrepancies and inaccuracies to occur in administrative data, which may make difficult certain cross-province comparisons.

Another issue related to the variability of interpretations among coders is visible in diagnostic typing. Where it can be difficult to discern a most responsible diagnosis, coders must interpret documentation and the coding standards to find the most correct coding scenario. Some coders, when reflecting on assigning diagnostic types where they are uncertain, describe it as “where [coding] comes into a blur” [Interview 16] or “where the art comes in” [Interview 12]. One coder even described diagnostic typing as “a stab in the dark most days” [Interview 20], because the most responsible diagnosis was so rarely discernible from available documentation. In such scenarios, coders type diagnoses to the best of their ability by using the resources they have available and consulting with other coders. However, coders do not all use the same resources, nor do they seek answers to uncertainty in the same ways. One coder even reported never being unsure of what type to assign diagnoses and never questioning their decision [Interview 9]. Again, this represents an issue to which its extent remains unknown.

Where unable to provide specific or consistent details about their subjective coding indicated, some coders indicated that they “only code what [they] feel might be important or might have a bit of influence on how the patient is treated there” [Interview 11], referring to the type 3 diagnoses which coders are not mandated to code. For instance, some reported coding type 3s because they felt that it did not add extra time, as they were experienced and even knew common type 3 codes by heart. Others coded type 3s because they felt it helped to “flesh out the story” [Interview 4] for those who would be analyzing data downstream. Regarding her decision to code history of tobacco, one coder noted that she always captured smoking, “just because I’m an avid non-smoker,” [Interview 4] which speaks to the influence of personal values in the coding process.

### Quotas and expectations

Aside from variability, the presence of a quota added pressure to coding that may influence data quality. While all coders reported their ability to meet the quota (i.e., on average 25 charts per day), they also noted that its presence created challenges, specifically regarding their relationships with management. One participant did describe that, “The expectation is to always make a certain quota. I know there’s days where the coders can’t and it’s not uncommon to be called into the office if you’re not making your quota” [Interview 27]. Casual coders, who work at various sites toward month-end to help sites meet their monthly quotas, especially reported feeling pressured and having their productivity closely monitored. As they explained, “It’s certainly different being casual to working [full-time]. Having your regular job […] you are able to take more time into looking for the answer and really studying it… whereas when you’re casual you do feel a little bit pressured to get enough of them done” [Interview 8]. Thus, where coders face pressures of benchmarking, there exists the potential to focus on productivity and quantity over quality, which may result in a lack of attention to detail among coders. Despite management indicating that their focus was on quality, coders still reported feeling that “the most important thing now is quantity and not quality… How fast can you go and how fast can we finish?” [Interview 5].

Other factors that provided challenges to coding but that coders did not perceive as greatly influencing quality include problems with the legibility of scanned documents in electronic medical records, a lack of feedback from management regarding coding quality, and frustrations with software programs and layout of the work environment.

## Discussion

In this paper, we set out to identify the barriers to high quality coding of administrative health data, from the perspective of medical chart coders, which to our knowledge has not yet been done through qualitative inquiry in the Canadian setting. In completing this aim, we identified that the majority of barriers to high quality coding exist upstream in the data generation process, particularly at the level of documentation completed by physicians and other healthcare providers. This differs from our initial expectation that barriers to data quality existed at the level of coding and abstracting. As we completed interviews with coders based on the problem areas identified in the literature (e.g., under-reporting of diagnoses, lack of specificity in coding, errors in coding diagnosis types) [3, 29–33], we found that the issues identified were products of much wider systemic issues. This is consistent with the literature on physician documentation, which has consistently pointed to this as a limiting factor in the quality of administrative data [23, 33–35]. In light of our findings, we conclude that any interventions targeted only at the level coders will not fully overcome limitations to data quality, as quality coding depends on quality documentation. We do, however, plan to develop a typology of factors that influence coding variability and subjective decision making in situations where it must occur (e.g., due to a lack of clear documentation). By making this process more transparent, coders and their managers may be able to further standardize subjectivity (e.g., adopt the same resources, follow the same decision-making process).

A key finding from our study is the pivotal need to implement interventions that improve physician documentation, for its illegibility, unreliability, and incompleteness at times present challenges to the precise coding of conditions and procedures essential to achieving high quality health data. As Tang and colleagues (2017) have recently found, there remains a disconnect between the purpose of physician documentation to assist in clinical care, versus its secondary use downstream (i.e., coding, billing) [36]. The situation is further complicated by the lack of communication that exists between physicians and coders in many hospitals, thus limiting coders’ opportunities to gain further clarity on documentation, as well as the different terminologies employed by physicians and the coders [36]. Such barriers and complications may be improved by reviewing data collection, documentation, and coding at the systemic level (e.g., who is responsible for data quality?).

Within the existing structure of documentation, however, there are multiple forms of interventions which may bear positive effects. The use of electronic health records is one area with great potential for improving documentation. Cowan et al. (2007), for instance, found that dermatologic surgeons and residents showed improvements in accuracy when their notes were electronic versus dictated, with 5.77% of electronic notes showing at least one error, compared to 81% in dictated notes [37]. Another study, by van Walravan et al. (1999) found that among hospital physicians-in-training and their supervisors, electronic discharge summaries were shown to be more complete (79.5% vs. 68.2%, *p* = 0.01) and timely (79.6% vs. 57.0%, *p* < 0.001), compared to those that were dictated and transcribed [38]. Dinescu et al. (2011) also found that the completeness of discharge summaries could be improved by implementing an audit and 30-min feedback session with geriatric medicine fellows, as post-intervention fellows were 20% more likely to have complete discharge summaries (*p* < 0.001) [39]. The findings from these studies are important, as they suggest the possibility for remediating some of the issues specific to coding. One possible intervention might focus on securing the presence of “most responsible diagnosis” on discharge summaries, through providing a template, educating residents of the importance of this element, providing feedback to physicians on this document, or auditing discharge summaries for physicians who have been trained to include this item. Even if only partially successful, this intervention could result in increased efficiency among coders who would be relieved of time spent searching for this information, and higher quality information, as this diagnosis type would directly reflect the doctor’s diagnosis without the need for interpretation.

A known limitation of this study is its generalizability outside of the Canadian and — to some extent — Albertan setting. Healthcare administration and service delivery differs provincially and territorially across Canada, so we recognize that the process in Alberta may not represent those in other jurisdictions. We do, however, believe that the barriers we found to high data quality will be comparable across jurisdictions, as reflective of wider systemic issues. One difference that may occur between coding in Canada and other countries regards training and education. In our study, we did not find that training provided any significant barrier to high quality coding among the coders we interviewed. We suggest this reflects the state of coding in Canada as a certified profession. Coder training in this country is regulated at the provincial and regional levels, and education programs exist at community colleges, universities, and through the Canadian Healthcare Association [40]. All programs are accredited by the Canadian College of Health Information Management (CCHIM) and require the successful completion of a national certification exam. To maintain certification, coders must participate in continuing medicinal education and register with the Canadian Health Information Management Association (CHIMA), which supports education, leadership, professionalism, and networking in the profession [41].

Other countries, however, do not have the same standards and training requirements, thus barriers to coding high quality information may differ and be more influenced by limitations in coder training. In England, for example, coding is carried out by non-clinical office staff, which makes the practice of abstracting more reliant on physician documentation [42]. In Thailand, the role of coding varies depending on the availability of staff at each site. Professionally trained, full-time coders do exist in Thailand; however, not at all locations. At smaller sites with higher staff turnover, coders included general office staff, medical statisticians, and nurses, whose coding would be overseen by a senior physician [43]. Unfortunately, these practices worked against sites’ ability to submit high quality diagnoses and procedure codes [43].

In contrast, the quality of administrative data in Canada is very high [44], despite the barriers and challenges to coding that exist, as documented in this paper. A re-abstraction study conducted by CIHI on 2009–2010 data found that significant diagnoses reported in the DAD were also found present in the chart 84.4% of the time [33]. Some significant conditions (i.e., ischemic heart diseases), were coded correctly up to 96% of the time [33]. It is likely that these findings reflect the standards of professional training and coding rules of classifications by which Canadian coders must abide. This study may therefore be of use to those who work to further improve data quality in the Canadian setting, such as trainers and decision-makers.

Following coding and abstraction, data is accessed for research, analysis, reporting, and public health surveillance. By the time that data reaches its users, it has already been cleaned by data custodians, who assess its quality based on the presence of certain items, duplication of others, and by ensuring the correct health identification number. In our observation, researchers operate under the assumption that the data they receive (in anonymized format) is of high quality and standardized. As such, data users may not fully understand the limitations of their data (e.g., miscoding, non-specific diagnoses, overrepresentation of certain conditions). This may influence the interpretation of their findings, which may even result in the misrepresentation of the prevalence of certain conditions or procedures. We intend for this work to contribute to data users’ understanding of the process through which coders translate health information into coded data, through identifying some of the barriers that coders face throughout this process.

As a final point, we believe this study comes at a turning point in the field of coding and abstraction, where coding is increasingly evolving towards the uptake of electronic medical records. At present, there still remains a need for careful coding and abstracting, as coders remain an essential element in the abstraction of Canadian health information. Additionally, there is also the need to resolve issues that influence data quality where possible. We suggest, however, that future research focus on the challenges that electronic medical records may introduce to the quality of administrative data, as well as the role that coders may play in ensuring this.

## Conclusions

In sum, coding is a complex process that is further complicated by the issues that exist throughout the process of administrative data generation. Inadequate, incomplete, non-specific, or imprecise documentation at the level of healthcare providers introduces uncertainty for coders, which may result in unspecified diagnoses. At the level of chart organization, incomplete charts were identified as a major barrier to data quality, which was driven by the pressure of a fast turnaround time to submit health information to the provincial clearinghouse. For coders, the main barriers to data quality stemmed from documentation issues that impeded their diagnosis typing. Additionally, the pressures of benchmarking introduced a quickened work pace in which limited coders’ attention to detail and their ability to re-check charts coded from incomplete documentation. Finally, the data users we spoke to did not seem to understand the process of administrative health data generation or the limited role that coders played in ensuring its quality. We hope this work has helped to illustrate this complex process and identified potential barriers to high quality coding, and that it will be of interest to healthcare providers, coders, researchers, analysts, and decision-makers who are concerned with its quality.

## Additional file

Additional file 1: Overview of process of administrative data generation in Canada. (DOCX 17 kb)
Additional file 2: Interview guide. (DOCX 25 kb)
Additional file 3: Table S1. Key differences in data elements of NACRS and DAD administrative databases. (DOCX 13 kb)

## Abbreviations

BIBA: Brought in by ambulance
CCHIM: Canadian College of Health Information Management
CHIMA: Canadian Health Information Management Association
CIHI: Canadian Institute for Health Information
DAD: Discharge Abstract Database
ICD: International Statistical Classification of Diseases and Related Health Problems
NACRS: National Ambulatory Care Reporting System
