# Supplementary material for: Barriers to data quality resulting from the process of coding health information to administrative data: a qualitative study
Source: BMC Health Serv Res. 2017 Nov 22;17:766. doi: 10.1186/s12913-017-2697-y (PMC5700659; doi:10.1186/s12913-017-2697-y)
Supplement: Additional file 1: — Overview of process of administrative data generation in Canada. (DOCX 17 kb) [file 12913_2017_2697_MOESM1_ESM.docx]

**Additional File 1: Overview of Process of Administrative Data Generation in Canada**

The process of administrative data generation begins when a patient is first seen at a healthcare facility to receive care. In Canada, professional coders are required to code demographic information, conditions, and procedures on all hospitalizations to generate DAD [1], and information from emergency department visits, day procedures, and acute care clinic visits to populate the National Ambulatory Care Reporting System (NACRS). At the point of arrival, a record is created or retrieved by an admissions clerk, who collects basic demographic information from the patient, including their unique health identification number. Aside from demographics, information is also collected about how the patient arrived at the healthcare facility (e.g., by ambulance). Patients seen in ambulatory care settings, such as emergency departments, day surgery clinics, and outpatient clinics have shorter and less complicated visits than patients who are admitted to hospital as inpatients. Inpatients spend more time in hospital and tend to have more complicated conditions, which results in greater interaction with healthcare providers. Some of the main differences in the information that is collected in these two settings are summarized in Supplementary Table 1, which highlights the differences between information collected for the DAD (inpatient) and NACRS (outpatient).

Throughout a patient’s time in the healthcare facility, events are documented by multiple healthcare providers (e.g., nurses, physicians, dieticians, occupational therapists). Following a patient’s discharge, the most responsible physician electronically writes out a narrative discharge summary or dictates one to be later written up by a medical transcriptionist. This summary is then attached to the front of a patient’s chart. At this point the chart, which now contains documentation about the patient’s visit, moves to the health records department where technicians ensure that all documentation is present and organized according to the preference of the unit from which the patient was discharged. The chart is then transferred to a centralized area to be retrieved by coding specialists.

Abstracting, that is “the process in which a human manually searches through a medical record to identify data required for secondary use” [2], is the primary role and responsibility of coders in Canada. Coders review documentation to abstract medical concepts for eventual submission to its health authority (e.g., Alberta Health Services), provincial Ministry of Health (e.g., Alberta Health), and CIHI. They abstract information according to a standard classification system (e.g., International Classification of Diseases, 10^th^ Revision) and follow the guidelines that CIHI provides for assigning codes for the diagnoses, procedures, and resources that are used throughout the encounter, in addition to also capturing administrative details (e.g., hospital number for reporting purposes). In Canada, up to 25 diagnoses and 20 procedures can be coded for each patient encounter. Coders assign a diagnosis type to indicate its significance and timing (discussed below). Length of stay and diagnosis are then used to calculate the resources used in hospital and to group patients into disease groupers and casemix groups [3].

Documentation from patient encounters is created by multiple healthcare providers representing many disciplines, which results in various forms of documentation that record different aspects of the patient’s stay. Some of the most common forms that coders use are the discharge summary or ambulatory care record, physician progress notes, nurses’ notes, physician operative reports, laboratory reports, x-rays, and patient history.

The documentation generated throughout a patient’s stay comprises their medical chart, which is either completely paper-based, completely electronic, or – most commonly – a hybrid of both. Abstracts are generated for the DAD or NACRS using multiple software programs that help specify conditions and locate codes. There are a large number of software programs that are used for abstracting and for electronic medical records that are not consistent across Alberta, or even across departments within a single healthcare facility. Software programs work in tandem, for a coder would first open an electronic medical record to access chart documentation (e.g., pathology reports, nursing notes) while also maintaining the paper chart in front of them, if applicable. Next, coders use an abstracting software to translate chart information into data, which prompts coders to input information from the chart about admission, discharge, procedures, conditions, and other details to generate an abstract. Coders are required to input detail to further specify conditions and procedures until a code is identified.

Once patient charts have been coded and abstracted, they are returned to the health records department until they are required at a later date for a new encounter or for coders to consult. Coders send their abstracts onto analysts at their local health record departments, who check the data for quality (e.g., admission time, duplicates), return them to coders for correction, and retain them for their own purposes (e.g., program planning) or to be accessed by researchers. Once the data have been corrected, they are submitted by coding managers to CIHI. CIHI also flags errors related to data quality and code accuracy (e.g., coding decision pathways that are not obviously apparent) and returns them to coders for correction. Following this, they aggregate and standardize the data and produce surveillance reports for participating institutions. At this point, researchers can request access to aggregate data from CIHI in its anonymized format for analysis.

**References**

1. Canadian Institute for Health Information, *Clinical administrative databases: Privacy impact assessment*, CIHI, Editor. 2012: Ottawa, ON, Canada.
2. Nahm, M., *Data accuracy in medical record abstraction*, in *School of Biomedical Informatics*. 2010, University of Texas Health Science Center at Houston: Houston, TX.
3. Lucyk K, L.M., Sajobi T, Quan H, *Disease Groupers: What are they, how are they used, and how do they compare internationally?* Perspectives in Health Information Management, 2016. **Summer**: p. 1-10.
