# Supplementary material for: Barriers to data quality resulting from the process of coding health information to administrative data: a qualitative study
Source: BMC Health Serv Res. 2017 Nov 22;17:766. doi: 10.1186/s12913-017-2697-y (PMC5700659; doi:10.1186/s12913-017-2697-y)
Supplement: Additional file 2: — Interview guide. (DOCX 25 kb) [file 12913_2017_2697_MOESM2_ESM.docx]

**Interview guide - CODERS**

Barriers to high quality coding of hospital chart information to administrative data:
A qualitative study

**PREAMBLE TO INTERVIEW**

“Hello. Thank-you so much for agreeing to participate in our study. We are interested in finding out more about the process of coding information from chart information to codes used in administrative data. Specifically, **we are interested in understanding the experiences of professional coders**, what is involved in this profession, and what difficulties professional coders face when trying to code chart information.

I will be **recording this session and this will then be transcribed into a written document to be analyzed**. We will keep the audio-recordings in a locked cabinet in an office accessible only to our study team. The text documents will be stored on a password-protected computer, also in an office accessible only to our study team.

Your information will be kept confidential and anonymous, where you will be assigned a study number, and transcriptions of this interview and any subsequent written documents will include only this study number without your identifying information. Any presentations or publications that result from the study will use study numbers only, again without any identifying information. At any time during this interview or afterward, you can choose to withdraw from the study. In this scenario, we would permanently destroy all the information that you have given to us, including your demographic details, the audio recording, transcripts of the recording, and any analysis of your data.

The interview will take about one hour. I have a rough outline here about some questions that we will cover, but please feel free to add any comments at any time. The topics that we will cover include:

1. Job training
2. The coding process
3. Work environment and collaboration

Are you ready to begin?”

**INTERVIEW GUIDE**

“First, let’s start with your **day to day work** as a professional coder.”

1. Tell me what a typical day looks like for you at work.

*Probes:*

1. How many charts do you code in a day?
2. How long does it take to code one chart?

1. Tell me about the **training** you have completed to prepare you to become a professional coder.

*Probes:*

- 1. Are there opportunities for continuing education that can help you to continue to improve your knowledge and skills?
     1. What about resources for self-learning and self-improvement?
     2. What about opportunities to learn from colleagues?
  2. Are there certain requirements you are required to have and maintain?

1. Are there areas in your job where you feel that your training did not prepare you for?

*Probes:*

- 1. What could be changed about your job training to better prepare you to be a professional coder?

“I’d now like to ask you a few questions about the **process of coding** charts and some areas where there might be room for improvement that you have found through your work.”

1. Tell me about the process you use to code each chart.

*Probes:*

1. Are there certain documents that you go to first or use the most?
2. How do you decide which diagnostic type to assign to a diagnosis?
   1. How do you decide how to code the most responsible diagnosis?
   2. In practice, how do you decide to apply a Type 2 diagnosis type to a certain diagnosis?
   3. Can you give me a recent example of a how you determined a certain diagnosis was a Type 2 diagnostic type?
3. What do you do if you are unsure what diagnostic type to assign to a diagnosis code?
4. How do you decide how many diagnoses to include?

a. Are there any challenges you face when trying to trying to follow the Canadian Institute for Health Information’s (CIHI) coding standards in assigning diagnosis type?

1. How do you decide how much detail to code?
2. What resources or references do you use to help you with the coding?

1. What could be improved in the hospital charts to help you with your coding?

*Probes:*

- 1. In the completeness or organization of the chart?
  2. To help you better read or understand the documents in the chart?

1. When coding chart information, what are some difficulties that you face that are related to the ICD-10 classification?

*Probes:*

What challenges do you face in:

1. Trying to assign codes that are as specific as possible?
2. Making sure your codes accurately reflect the diagnoses documented in the chart?

7. Are there challenges you face when switching between coding systems?

1. What do you do when you are uncertain about information in hospital charts or if information is missing?

*Probes:*

- 1. Do you encounter this often?

9. What is your understanding of what happens to chart information after you code it?

“I also want to ask you about your **work environment** and how that affects your day-to-day work.”

1. Tell me about your work environment.

*Probes:*

- 1. How does your physical space help (or hinder) your coding?
  2. How does collaboration with others help with your coding?
  3. How does having a coordinator (or coding manager) help with your coding?
  4. How are you given feedback about your coding? Does it help?
  5. How does the computer software that you use to code hospital charts help or hinder your coding?

1. How can your work environment be improved to help you with your coding?
2. How do you feel about your workload and how it affects your coding?

“We are almost at the close of our interview, so I wanted to give you the opportunity to **share anything else** with me about the experiences and challenges you have had with coding chart information.”

1. Is there anything else you would like me to know?

“Finally, before we finish I need to collect a few demographic details from you.”

1. How old are you?
2. How long have you been a professional coder?
3. Do you work full time or part time?
4. What training did you undergo to become a coder? *(If not addressed previously)*
   1. *Level of education – post-secondary, university, high school, etc.*
5. What is your job title?
6. Do you belong to any professional associations (e.g., CHIMA, AHIMA)?

“Thank-you very much for taking time to meet with me and complete this interview. If you have any questions or concerns, please email me at <klucyk@ucalgary.ca>. Thank-you again. Good-bye.”
