# Supplementary material for: Barriers to data quality resulting from the process of coding health information to administrative data: a qualitative study
Source: BMC Health Serv Res. 2017 Nov 22;17:766. doi: 10.1186/s12913-017-2697-y (PMC5700659; doi:10.1186/s12913-017-2697-y)
Supplement: Additional file 3: Table S1. — Key differences in data elements of NACRS and DAD administrative databases. (DOCX 13 kb) [file 12913_2017_2697_MOESM3_ESM.docx]

**Additional file 3: Table S1. Key differences in data elements of NACRS and DAD administrative databases.**

|  | **NACRS** | **DAD** |
| --- | --- | --- |
| **Admission** | “Arrival and visit type”  Ambulatory visit status, mode of patient contact, arrival date and time, registration date, registration time, referral source, referral date, institution transferred from, presenting complaints (symptom, complaint, problem, reason), whether visit was arranged or ED visit | “Admission”  Admission date/time, Institution admitted from (if applicable), status of patient at time of admission, admit via ambulance, when patient left ED (if applicable) |
| **Demographic Data** | ““Patient/Client Demographic Data”  Health care number, Provincial/territorial health care number, responsibility for payment, postal code, Gender (sex), birth date, access to primary health care | “Patient/Client Demographics”  Health Care Number (provincial/territorial), Postal code, Gender (sex), responsibility for payment, provincial/territorial healthcare number, birthdate |
| **Arrival and visit type** | “Arrival and visit type”  Mode of visit, arrival date and time, registration visit date and time, institution from (if applicable, referral date), ED visit indicator | “Admission”  Admission date and time, institution from (if applicable), status of patient at time of admission, point of entry into facility, admit via ambulance, time spend in ED (if applicable) |
| **Provider information** | Provider type, provider service, provider number, program area | Provider type, provider number, provider service |
| **Patient service** | N/A | Main patient service or subservice, Service transfer, weight (of newborn or neonate), service or subservice transfer and transfer days |
| **Diagnosis information** | “Assessment and consultation data”  Main problem, other problem | “Diagnosis information”  Diagnosis, diagnosis type |
| **Intervention Data** | Main intervention/other intervention, main intervention/other intervention attributes (details), duration of abulatory care intervention, intervention location, anaesthetic technique, main intervention and/or other intervention start date and time , out-of-hospital indicator, out-of-hospital institution number | Intervention, Intervention start date and time, intervention attributes (status, location, extent), intervention provider number (not in AB), intervention provider service, intervention location, anesthetist,anesthetic technique, out-of-hospital indicator, out-of-hospital institution, unplanned return to intervention location, died during intervention, intervention end date and time, intervention pre-admit flag (if applicable) |
| **Separation** | Status of patient on discharge (disposition), institution transferred to, date and time when decision about patient’s disposition was made, date and time left ED | Discharge date and time, institution transferred to, status of patient on discharge |
| **Submission control elements** | Facilitiy’s province/territory, facility’s ambulatory care number, submission fiscal year, submission period, abstract number, **coder number**, chart number, ambulatory registration/encounter, submission level code | Institution number, batch count, batch year, batch period, batch number, abstract number, **coder number**, chart number, maternal/newborn chart number |
| **Special flags related to data quality** | 108 – A flag to identify when data was collected from an incomplete record (optional) | 07/04 – An indicator that not all data can be accommodated on the abstract (optional) |
